# Improving Knowledge on Pathways to Functional Outcome in Schizophrenia: Main Results From the Italian Network for Research on Psychoses

**DOI:** 10.3389/fpsyt.2021.791117

**Published:** 2021-12-14

**Authors:** Luigi Giuliani, Giulia Maria Giordano, Paola Bucci, Pasquale Pezzella, Francesco Brando, Silvana Galderisi

**Affiliations:** Department of Psychiatry, University of Campania “Luigi Vanvitelli”, Naples, Italy

**Keywords:** schizophrenia, real-life functioning, recovery, neurocognition, social cognition, negative symptoms

## Abstract

The identification of factors associated with functional outcome of subjects with schizophrenia is a great challenge in current research oriented to the personalization of care. The Italian Network for Research on Psychoses (NIRP) is a network of 26 university psychiatric clinics and/or mental health departments aimed to carry out multicenter research projects to improve the standards of prevention, diagnosis, and treatments of schizophrenia. The network has promoted 2 main studies, a cross-sectional one and a longitudinal one and seven “add-on” studies. The cross-sectional study of the network included 921 subjects with schizophrenia, 379 unaffected first-degree relatives of these patients, and 780 healthy controls. Results from this study documented that social and non-social cognition, functional capacity, negative symptoms, resilience, and family or social incentives strongly influence a measure of global functioning. The follow-up study included 618 patients from the original sample and has produced evidence of the key role of cognition, functional capacity, the experiential domain of negative symptoms, and everyday life skills in predicting functional outcome. The longitudinal study demonstrated that social cognition and the experiential domain of negative symptoms had an impact on interpersonal functioning, while non-social cognition had an impact on everyday life skills. Both non-social cognition and social cognition predicted work skills. The research question concerning the relationships of cognitive impairment and negative symptoms has been investigated with an innovative approach, using a structural equation model (SEM) and a network analysis. Both analyses demonstrated that only the experiential domain of negative symptoms had a distinct direct effect on functioning. The network analysis showed that expressive deficit was connected to functional capacity, as were social and non-social cognitive variables, and to disorganization. These findings were confirmed by the follow-up study. The add-on studies showed distinct electrophysiological correlates of the two negative symptom domains and the partial overlap between disorganization and neurocognitive impairment. Moreover, they identified and characterized a specific subgroup of patients suffering from schizophrenia with autism spectrum symptoms. The NIRP studies have implications for personalized management of patients with schizophrenia and highlight the need for a careful assessment of several domains rarely evaluated in clinical settings.

## Introduction

Schizophrenia is a severe mental disorder that presents a high heterogeneity in terms of risk factors, clinical manifestations, comorbidities, treatment response, and outcomes (1–19).

About 75% of people suffering from this disorder shows a clinical course characterized by relapses and remissions (20–23), and <1 in 7 people meets the criteria for recovery (24, 25). Two aspects are fundamental to achieve clinical recovery in schizophrenia: the remission of symptoms and the improvement in functioning (26–29). However, despite the introduction of innovative pharmacological and psychosocial treatments that facilitate symptomatic remission (3, 6–8, 10), the impairment in different areas of real-life functioning still represents an unmet need in the care of people suffering from schizophrenia, thus causing a huge burden on patients, their families, and health care systems (30–41).

A variety of factors, some related to the illness, some to personal resources, and others to the social context, seem to influence functional outcome, through direct or indirect relationships (18, 41–50). The identification of these factors, as well as their relative impact on the outcome through complex pathways, represents, to date, a main goal of current psychiatric research, in order to develop integrated and individualized treatments aiming at ameliorating functioning and thus at achieving recovery (51, 52).

Within this frame, a national multicenter project, promoted by the Italian Network for Research on Psychoses, has been developed. This is a network of 26 university psychiatric clinics and/or mental health departments, coordinated by the Department of Psychiatry of the University of Campania “Luigi Vanvitelli” (Table 1). By promoting and enhancing the collaboration among the involved centers, this network is intended to carry out research projects in order to improve the standards of prevention, diagnosis, and treatments for people suffering from primary psychotic disorders. So far, the network promoted two main studies, a cross-sectional one and a longitudinal 4-year follow-up one. In addition, seven “add-on” studies have been promoted by the network (Table 2).

**Table 1:** Centers involved in the Italian Network for Research on Psychoses and their coordinators.

**Center**	**Coordinator**
Department of Psychiatry, University of Campania “Luigi Vanvitelli”	Silvana Galderisi
Department of Neuroscience, Section of Psychiatry, University of Turin	Filippo Bogetto/Paola Rocca
Department of Translational Medicine, Psychiatric Unit, University of Eastern Piedmont	Patrizia Zeppegno
Department of Psychiatry, State University of Milan	Carlo Altamura
Psychiatric Unit, School of Medicine, University of Brescia, Brescia	Emilio Sacchetti/Antonio Vita
Department of Neurosciences, Rehabilitation, Ophthalmology, Genetics and Maternal and Child Health, Section of Psychiatry, University of Genoa	Mario Amore
Department of Neurosciences, Psychiatric Clinic, University of Padua	Paolo Santonastaso/Angela Favaro
Department of Biomedical and Neuromotor Sciences, University of Bologna	Diana De Ronchi
Department of Neuroscience, Psychiatry Unit, University of Parma	Carlo Marchesi
Department of Neurosciences, Psychology, Drug Research and Child Health, University of Florence	Stefano Pallanti
Department of Health Sciences, Psychiatry Unit, University of Florence	Valdo Ricca
Department of Clinical and Experimental Medicine, Section of Psychiatry, University of Pisa	Liliana Dell'Osso
Department of Molecular Medicine and Clinical Department of Mental Health, University of Siena	Andrea Fagiolini
Department of Life, Health and Environmental Sciences, Unit of Psychiatry, University of L'Aquila	Massimo Casacchia/Rita Roncone
Department of Biotechnological and Applied Clinical Sciences, Section of Psychiatry, University of L'Aquila	Alessandro Rossi
Department of Neuroscience and Imaging, G. D'Annunzio University of Chieti	Massimo di Giannantonio
Department of Neurology and Psychiatry, Sapienza University of Rome	Massimo Biondi
Department of Neurosciences, Mental Health and Sensory Organs, S. Andrea Hospital, Sapienza University of Rome	Paolo Girardi/Maurizio Pompili
Department of Systems Medicine, Psychiatry and Clinical Psychology Unit, Tor Vergata University of Rome, Rome	Alberto Siracusano
Department of Neuroscience, Reproductive Science, and Odontostomatology, Section of Psychiatry, Federico II University of Naples	Andrea De Bartolomeis
Department of Medicine, Surgery and Dentistry “Scuola Medica Salernitana,” Section of Neuroscience, University of Salerno	Palmiero Monteleone
Department of Neurological and Psychiatric Sciences, University of Bari	Alessandro Bertolino
Department of Clinical and Molecular Biomedicine, Psychiatry Unit, University of Catania	Eugenio Aguglia
Department of Public Health, Clinical and Molecular Medicine, Section of Psychiatry, University of Cagliari	Bernardo Carpiniello
Psychiatry Unit, Department of Medical Sciences, University of Foggia	Antonello Bellomo
Department of Psychiatry, Neurobiology, Pharmacology and Biotechnologies, UNIPI	Mauro Mauri

**Table 2:** Add-on studies of the Italian Network for Research on Psychoses.

**Add-on studies**
Investigation of electrophysiological correlates of schizophrenia and their association with psychopathology, social and non-social cognition, and real-life functioning
Investigation of structural–functional magnetic resonance imaging features associated with diagnosis and real-world functioning in patients with schizophrenia
Investigation of autistic spectrum symptoms and their impact on real-life functioning in subjects with schizophrenia
Investigation of sexual functioning in subjects with schizophrenia and its association with psychopathology and social functioning
Investigation of obsessive symptoms and their impact on real-life functioning in subjects with schizophrenia
Investigation of resources and global burden of patients' families and their impact on psychopathology and real-life functioning of subjects with schizophrenia
Investigation of post-traumatic spectrum symptoms and their impact on real-life functioning in subjects with schizophrenia

The cross-sectional study had been carried out between 2011 and 2013 (32, 33). The primary objective of the study was to identify factors affecting real-life functioning of subjects with schizophrenia and to define their relative contribution. The longitudinal study was conducted after 4 years. This study investigated whether factors identified as predictors and mediators of real-life functioning in the cross-sectional study were confirmed as such as follow-up (34, 37). As compared to previous studies on the topic, both studies analyzed a greater number of variables, some of which have never been examined before. Moreover, these studies used state-of-the-art instruments for the assessment of each variable included and appropriate data analysis methods in order to explore the complex relationships between possible predictors, mediators, and outcome measures.

The implementation of the longitudinal assessment allowed us to overcome the limitations of the cross-sectional design, which prevented inferences about the direction of causality. In fact, the majority of studies investigating factors associated with functional outcome in schizophrenia have had a cross-sectional design, while only few and inconsistent findings have been reported by investigations with a longitudinal design (44, 53–63). The inconsistency of results might be due to different factors, such as the small sample sizes included in the studies, the use of different measures of functional outcome, and the use of assessment instruments, especially for cognitive impairment and negative symptoms, that were often not in line with their current conceptualization (32, 64–67). Indeed, although negative symptoms and cognitive impairment are stable dimensions of schizophrenia, are often present since the early phases of the illness, persist into clinical remission, and predict outcomes (41, 56, 57), uncertainties still remain about the correct evaluation and management of these dimensions (4, 68–70).

In the present article, we report the main findings of the two studies conducted by the network, which have contributed to the advancement of knowledge on the complex pathways involved in functional outcomes in people with schizophrenia.

## Cross-Sectional Study

### Participants

Within the cross-sectional study, 921 patients with a diagnosis of schizophrenia, aged between 18 and 66 years; 379 unaffected first-degree relatives of these patients; and 780 healthy controls were recruited (32, 33). For the patient group, inclusion criteria were a diagnosis of schizophrenia according to DSM-IV, confirmed with the Structured Clinical Interview for DSM-IV–patient version (SCID-I-P), and an age between 18 and 66 years. Exclusion criteria were a history of head trauma with loss of consciousness; a history of moderate to severe mental retardation or of neurological diseases; a history of alcohol and/or substance abuse in the last 6 months; current pregnancy or lactation; inability to provide an informed consent; and treatment modifications and/or hospitalization due to symptom exacerbation in the last 3 months. For each recruited patient who agreed to involve relatives, two first-degree relatives were recruited, when available. They were preferably the two parents, or one parent and one sibling, or two siblings. These relatives were included in the study if criteria for a current or lifetime psychiatric diagnosis were not met when they were interviewed with the SCID-I–non-patient version and the SCID-II. Exclusion criteria were (a) a history of head trauma with loss of consciousness; (b) a history of moderate to severe mental retardation or of neurological diseases; (c) a history of alcohol and/or substance abuse in the last 6 months; (d) inability to provide an informed consent. Healthy subjects matched with patients for gender and geographical area of origin were recruited from the community at the same sites as the patient sample. Inclusion and exclusion criteria were the same as those listed for first-degree relatives.

### Assessment Instruments

The study evaluated the impact on real-life functioning of a larger number of variables compared to previous investigations, some of which had never been investigated before. The assessed variables were grouped into three categories: (a) illness-related variables (positive, negative, disorganized, depressive, and extrapyramidal symptoms; neurocognition; social cognition; and functional capacity); (b) personal resources (resilience and engagement with mental health services); and (c) context-related factors (socio-demographic variables; socioeconomic status; availability of a disability pension; access to family and social incentives; and social network). The variables included in each category are reported in Table 3. Real-life functioning was chosen as any index of clinical recovery. State-of-the-art instruments were used to assess variables of each category and real-life functioning. The adopted instruments were chosen on the basis of the literature and the researchers' experience, to overcome limitations of previous studies. When it was necessary, assessment instruments were translated, adapted, and validated for the Italian context.

**Table 3:** Investigated variables in the cross-sectional and follow-up studies of the network.

**Factors**	**Variables**	**References**
Illness-related variables	Neurocognitive deficit	(71–73)
	Social cognition deficit	(74, 75)
	Negative symptoms	(66, 70, 76–81)
	Depressive symptoms	(78, 82, 83)
	Positive symptoms	(42, 50, 84)
	Disorganization	(50, 85)
Personal resources	Resilience	(49, 86)
	Service engagement	(87)
Context-related variables	Social network	(45, 46)
	Job or housing opportunities and residential support	(45, 46)
	Disability compensation	(45, 46)
	Internalized stigma	(48, 88)

All the instruments have been used to evaluate subjects with schizophrenia, their first-degree relatives, and healthy controls.

#### Illness-Related Variables

A clinical form was filled in with data on age of disease onset, course of the disease, and treatments received, using all available sources of information (patient, family, medical records, and mental health workers).

The severity of positive and disorganized symptoms was evaluated using the Positive and Negative Syndrome Scale (PANSS) (89).

Negative symptoms were assessed with the Brief Negative Symptom Scale (BNSS), a second-generation rating scale which is in line with the current conceptualization of negative symptoms (64, 90). As compared to first-generation rating scales, the BNSS shows several advantages. It does not include aspects that are related to cognitive or depressive dimensions; it provides a separate assessment of behavior and inner experience for items referring to experiential deficits such as avolition, thus enabling a better differentiation from social functioning and other subjective experiences such as decreased interest or energy; it provides a separate assessment of consummatory and anticipatory anhedonia; it generates a total score as well as separate scores for the five negative symptom domains (avolition, anhedonia, asociality, blunted affect, and alogia). The two-factor structure, consisting of the experiential domain (avolition, anhedonia, and asociality) and the expressive deficit domain (blunted affect and alogia), is supported by the use of this instrument (64, 66, 90–92). Depressive symptoms were evaluated using the Calgary Depression Scale for Schizophrenia (CDSS) (93); extrapyramidal symptoms, with the St. Hans Rating Scale (SHRS) (94). The Measurement and Treatment Research to Improve Cognition in Schizophrenia (MATRICS) Consensus Cognitive Battery (MCCB) was adopted to evaluate cognitive impairment (65, 67). This instrument was built up within the MATRICS initiative, aiming to develop a cognitive battery for subjects with schizophrenia designed for use in clinical trials (65, 67). MCCB assesses seven cognitive domains that are reported to be compromised in subjects with schizophrenia: speed of processing, attention and vigilance, working memory, verbal learning and memory, visuospatial learning and memory, reasoning and problem solving, and social cognition (44). The MCCB Mayer-Salovey-Caruso Emotional Intelligent Test (MSCEIT), the Facial Emotion Identification Test (FEIT) (95), and the Awareness of Social Inference Test (TASIT) (96) were used to measure different aspects of social cognition, such as emotional intelligence, emotion recognition, and theory of mind.

#### Personal Resources

The Resilience Scale for Adults (RSA) (97) was used to assess resilience; the Service Engagement Scale (SES) (87), to evaluate the access of subjects with schizophrenia to mental health services.

#### Context-Related Factors

A socio-demographic questionnaire was developed *ad hoc* to collect data on gender, age, marital status, schooling, housing, eating habits, substance use, socioeconomic status, availability of a disability pension, and access to family and social incentives (32). The Internalized Stigma of Mental Illness (ISMI) (98) was used to assess stigma in subjects with schizophrenia.

#### Functional Capacity and Real-Life Functioning

The functional capacity was assessed through the brief version of the University of California, San Diego (UCDS) Performance-Based Skills Assessment–brief version (UPSA-B), a performance-based instrument that assesses “financial skills” and “communication skills” (99).

Real-life functioning was evaluated with the Specific Level of Functioning Scale (SLOF) (100, 101), a hybrid scale endorsed by the panel of experts involved in the Validation of Every-day Real-World Outcomes (VALERO) (100–103), which evaluates different areas of functioning and is based on the key caregiver's judgment on behavior and functioning of patients. The use of the SLOF allowed us to overcome limitations of previous studies investigating real-life functioning, which examined only a single or fewer domain(s) of functioning and collected only information from patients that could be influenced by many factors (e.g., delusions, hallucinations, lack of insight, disorganized thinking, cognitive deficits, or depression). The SLOF includes 43 items grouped into six domains: physical functioning, personal care skills, interpersonal relationships, social acceptability, everyday life skills, and work skills.

For each category of variables, at least one researcher per site was trained. In order to avoid halo effects, the same researcher could not be trained for more than one category. A good to excellent agreement among raters was observed for the instruments included in the study (32).

#### Translation and Validation of Assessment Instruments

The BNSS was translated in Italian and validated within the cross-sectional study (92, 104). The validation study showed an excellent inter-rater reliability and a good convergent and discriminant validity, confirming that BNSS is a reliable tool for the assessment of negative symptoms in multicenter studies.

The validation study of the Italian version of the SLOF showed a good construct validity and internal consistency and a well-delineated factor structure of the instrument (100, 105).

The MCCB was translated in Italian (106), and this version was validated in a large sample, composed by subjects with schizophrenia, their unaffected first-degree relatives, and healthy controls (107). Furthermore, in collaboration with the MCCB developers, the standardization of raw scores through the computation of T scores was performed, using the scores of the normative Italian sample (107).

The TASIT manual was translated and the related video clips were dubbed in Italian, at the Fono Roma Studio (www.fonoroma.com) (108). In addition, the Italian version of the FEIT was developed (108).

### Statistical Analysis

In order to investigate the simultaneous impact on functional outcome of multiple factors interacting with each other, two main statistical approaches were used: the structural equation model (SEM) and the network analysis. The SEM consists in a set of simultaneous multiple regression models for estimating and testing a pathway of relationships among variables (measured variables and latent constructs) (109). This approach allows researchers to infer causal relationship among predictors and outcome and to identify possible mediation and moderation factors, with the estimation of direct, indirect and total effects. It requires *a priori* assumptions of the possible associations among variables and of possible predictors, mediators, and outcomes, which is not always possible, especially because of the non-unidirectionality of some relationships (e.g., illness-related variables may influence real-life functioning and vice versa). In order to overcome these limits, a second approach, the network analysis, was used (110, 111). This type of analysis is a data-driven approach which does not require an *a priori* modeling of relationship among variables but generates a spatial ordered network where strongly related variables are at the center of the network and the weakly related ones at the periphery. Furthermore, estimating the number and the strength of variable connections and their closeness, this approach allows us to investigate which variables belong to the same construct and how different constructs are mutually interacting and reinforcing each other (111).

### Results From SEM and Network Analyses

**Figure 1:**
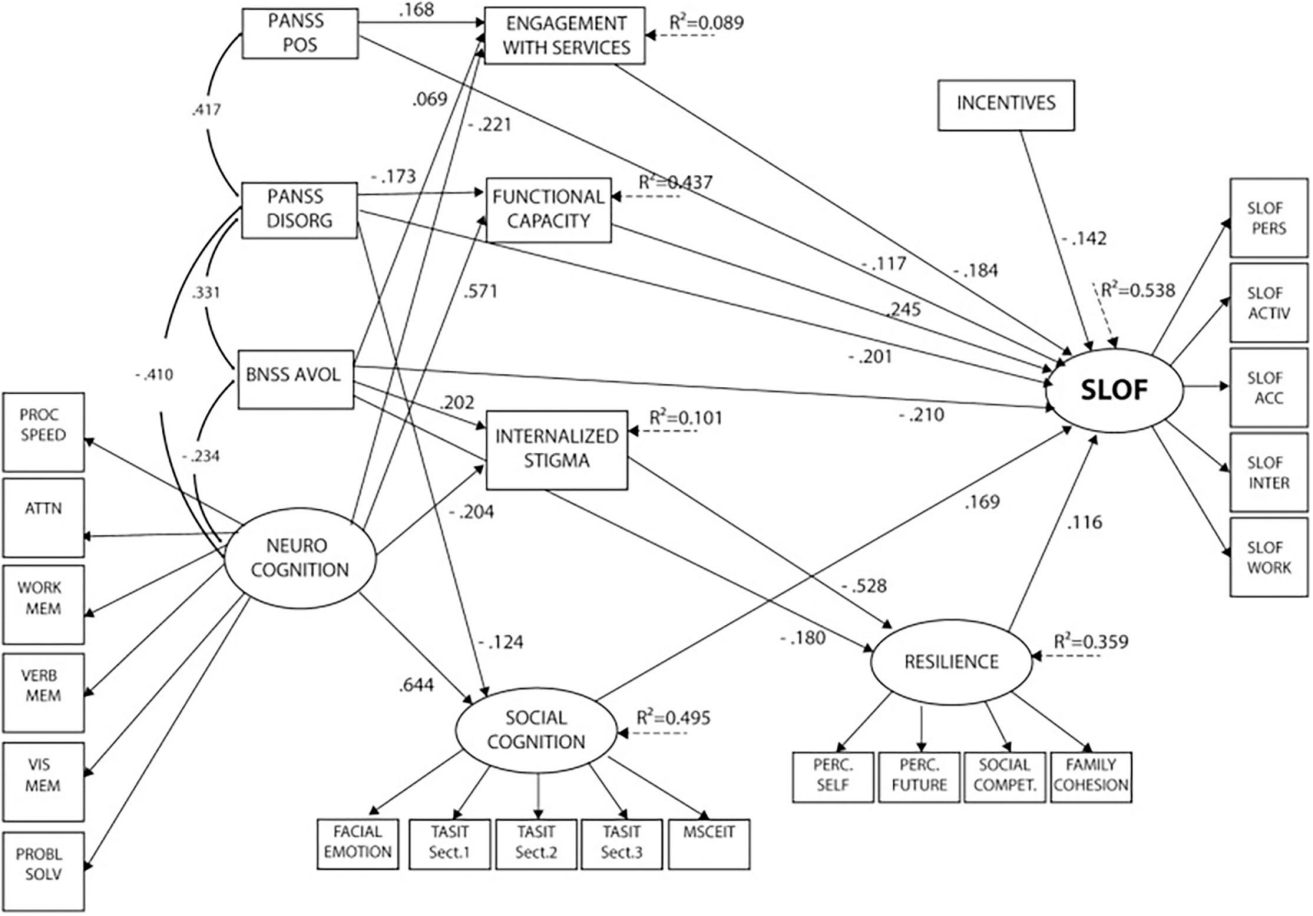
Final structural equation model after trimming of non-significant paths. Neurocognition, social cognition, resilience, and SLOF are latent variables (with arrows pointing to their respective indicators). PANSS POS, PANSS DISORG, BNSS avolition, neurocognition, and incentives are independent predictors. Social cognition, functional capacity, internalized stigma, resilience, and service engagement are mediators, and SLOF is the dependent variable. PANSS, Positive and Negative Syndrome Scale; POS, positive; DISORG, disorganization; BNSS, Brief Negative Symptom Scale; EE, poor emotional expression; AVOL, avolition; PROC SPEED, processing speed; ATTN, attention; WORK MEM, working memory; VERB MEM, verbal memory; VIS MEM, visuospatial memory; PROBL SOLV, problem solving; TASIT, The Awareness of Social Inference Test; MSCEIT, Mayer-Salovey-Caruso Emotional Intelligence Test; PERC. SELF, perception of self; PERC. FUTURE, perception of the future; SOCIAL COMPET, social competence; SLOF, Specific Level of Functioning; PERS, skills in self-care; ACTIV, community activities; ACC, social acceptability; INTER, interpersonal relationships; WORK, working abilities.

SEM analysis (32) showed that disorganization, the experiential domain of negative symptoms (including avolition, asociality, and anhedonia), positive symptoms, deficits in neurocognition, social cognition and functional capacity, internalized stigma, low resilience, and poor access to familial and social incentives had a significant direct and/or indirect impact on real-life functioning, explaining 53.8% of the variance. Neurocognition showed the strongest association with real-life functioning. The impact of neurocognition on the outcome turned out to be mainly indirect, mediated by functional capacity, social cognition, engagement with services, and internalized stigma. Social cognition also had a direct influence on real-life functioning, independently from neurocognition and negative symptoms. Service engagement was directly associated with the functional outcome, while internalized stigma showed an indirect impact on real-life functioning, mediated by resilience. Psychopathology, in particular positive and disorganized symptoms, and the experiential domain of negative symptoms were found to be directly and indirectly correlated with real-life functioning. The impact of positive symptoms was mediated by service engagement; the effect of disorganization, by functional capacity; and the impact of the experiential domain, by services engagement, internalized stigma, and resilience (see Figure 1).

The network analysis (33) confirmed that neurocognition, social cognition, resilience, and real-life functioning are well-defined independent constructs.

With respect to psychopathologic aspects, the experiential and expressive deficit domains of negative symptoms were highly interconnected but showed different associations. In particular, the experiential domain was associated with depression, social competence, “interpersonal relationships,” and “work skills,” while the expressive deficit was associated with disorganization, functional capacity, and “everyday life skills.” Depression did not show any connection with real-life functioning. Positive and disorganized symptoms had few connections to the other nodes and were peripheral nodes in the network.

The most central and interconnected nodes of the obtained network were functional capacity and everyday life skills.

Functional capacity was shown to be the bridge between cognition (neurocognition and social cognition) and real-life functioning, in particular with the “everyday life skills.” The neurocognition and social cognition constructs were adjacent and densely connected and interconnected. Both constructs had a high impact on functional capacity, and through this, on real-life functioning. In the social cognition domain, the TASIT-1, which measures the ability to identify basic emotions, showed the highest connection with functional capacity. This finding might suggest that a good comprehension of social and emotional stimuli may lead to a better acquisition of interpersonal skills required for some of the tasks incorporated in the functional capacity assessment (e.g., communication skills).

Furthermore, also the SLOF domain “everyday life skills” had a central position within the network and connected other real-life functioning domains with psychopathology, internalized stigma, functional capacity, and through this, neurocognition and social cognition.

### Other Results of the Cross-Sectional Study

In first-degree relatives of subjects with schizophrenia, similar direct or indirect interactions among predictors, mediators, and functional outcome were observed (112). These findings confirm the results of the main cross-sectional study, in the absence of confounding factors, such as residual psychotic symptoms and pharmacological treatment. In addition, the presence of impairment in the “interpersonal relationships” and “work skills” also in the group of unaffected relatives suggests the possible involvement of schizophrenia vulnerability factors.

The specific impact of personal resources on functional outcome has been investigated in three different network studies (113–115). A greater resilience and a higher degree of education were associated with a better social functioning, while worse problem solving and higher internalized stigma, along with male gender and depression, were associated with more severe symptoms (114). Furthermore, lower resilience, more severe negative symptoms, and female gender were associated with depressive symptoms, while internalized stigma represented a mediator between negative symptoms and resilience, suggesting a complex relationship between personal resources, negative symptoms, and depression in schizophrenia (115). The third study investigated the relationship between self-reported personal recovery and functional recovery, identifying three different clusters of patients: (a) patients with good personal recovery and good functional outcome; (b) patients with poor personal recovery and poor functional outcome; and (c) patients with intermediate personal recovery (between the other two clusters), with good insight and high levels of depression (113). These studies underline the importance and need of an accurate characterization of personal resources in subjects with schizophrenia, in order to implement individualized treatment plans aimed at improving different aspects of these resources, which have a different impact on functioning.

The role of social cognition and its impact on functioning were investigated in a study conducted by Rocca et al. (108). The authors identified three groups of patients: (a) patients without impairment in social cognition; (b) patients with a moderate impairment in social cognition; and (c) patients with a strong impairment in social cognition. This study revealed a linear relationship between social cognition, neurocognition, disorganization, and real-life functioning across the three groups. Positive symptoms were lower in patients without social cognition impairment, as compared to those with a moderate and a strong impairment in social cognition. Furthermore, negative symptoms were highest in subjects with moderate social cognition deficits, compared to subjects with absent or severe impairment in social cognition (108).

The relationship between disorganization and real-life functioning has emerged in one study that demonstrated that conceptual disorganization, among other disorganized symptoms, was the most relevant one impacting, through direct or indirect associations, “everyday life skills” (116).

Mucci et al. (107) reported impairment of all MCCB domains in subjects with schizophrenia. First-degree relatives showed a pattern of neurocognitive impairment, with intermediate scores between those of patients and healthy controls. In addition, patients' MCCB scores were able to predict the first-degree relatives scores on all domains except for visual learning.

One study investigated the role of premorbid academic and social functioning impairment on real-life functioning, cognition, and psychopathology. Subjects with schizophrenia showed an impairment in premorbid academic and social functioning compared to healthy controls, while first-degree relatives had only impairment in academic aspects (117). In patients, impairment of premorbid functioning predicted severity of negative symptoms, working memory deficits, social cognition deficits, and real-life functioning. These data suggest that a poor premorbid functioning might represent a vulnerability marker of schizophrenia and highlight the need to implement early psychosocial and cognitive remediation interventions (118).

One study explored the association between insight and depressive symptoms and reported greater self-depreciation, pathological guilt, morning depression, and suicidal ideation in patients with high levels of insight (119).

Another explored aspect was the prevalence of extrapyramidal symptoms in subjects with schizophrenia and its association with neurocognition, social cognition, and psychopathology. The network analysis showed that parkinsonism was directly connected to both psychopathological and neurocognitive indices, whereas no direct connection emerged between extrapyramidal symptoms and social cognition (120).

Two studies investigated also genetic aspects in this population (121, 122). In particular, one study investigated *de novo* copy number variations (CNVs) in the whole-genomic DNA obtained from 46 family trios of schizophrenia probands. The authors reported the presence of *de novo* CNVs in genes involved in brain and neural development, suggesting that these alterations could contribute to the genetic vulnerability to the disorder (122). The study by Gennarelli et al. (121) aimed to explore the genetic basis of social cognition, using a genome-wide study approach. The authors found significant associations between the patients' ability in social inference and the *TMEM7M4* gene.

## Longitudinal Study

### Participants

After 4 years, 618 subjects, out of the 921 subjects with schizophrenia enrolled in the cross-sectional study, agreed to participate in the longitudinal study (34, 37). These subjects did not differ from the rest of the baseline sample with respect to socio-demographic characteristics, illness-related factors, personal resources, context-related factors, and real-life functioning (34, 37). First-degree relatives and healthy controls were not recruited for the longitudinal study.

### Assessment Instruments

In order to evaluate illness-related variables, personal resources, context-related factors, and real-life functioning in subjects with schizophrenia after 4 years from the cross-sectional study, the same assessment instruments used at baseline were adopted.

In addition, in the longitudinal study, the patients' insight on their real-life functioning impairment, as well as the awareness of their own cognitive impairment in several domains, was also investigated. Therefore, the SLOF was administered to both patients and their caregivers, in order to explore their accuracy in self-reporting functioning. Moreover, the Cognitive Assessment Interview (CAI) was introduced in the longitudinal study (123). This is a second-generation co-primary measure and consists of 10 items that investigate six of the seven impaired domains in subjects with schizophrenia (concerning the visuospatial memory domain, no interview question was deemed appropriate). This instrument was administered to the patient and his or her caregiver to measure the perceived severity of the impairment in several cognitive domains. The impact of cognitive impairment on the patients' daily functioning, the patients' awareness of their own cognitive deficits, and the possible discrepancy between the patients' and caregivers' interviews were evaluated. The CAI was translated and adapted for the Italian context and showed a good to excellent reliability and excellent internal consistency (124).

### Statistical Analysis

In order to test whether variables affecting real-life functioning in the cross-sectional study confirmed their influence at follow-up and which variables were related to changes in real-life functioning at follow-up, SEM and latent change score (LCS) modeling were conducted, respectively. Moreover, a network analysis was used to investigate whether the pattern of relationships among variables involved in the cross-sectional study was similar at follow-up and to compare the network structure of recovered and non-recovered patients at follow-up. For the classification of recovered and non-recovered patients at the 4-year follow-up, we used two criteria: (1) the presence or absence of symptomatic remission according to the Andreasen criteria and (2) the presence or absence of functional recovery, defined as a weighted score of at least 76.2 on SLOF “interpersonal relationships,” “work skills,” and “everyday life skills” scales (34).

### Results From SEM and Network Analyses in the Longitudinal Study

In the longitudinal study, SEM and LCS analyses (37) showed that baseline measures of neurocognition, social cognition, the experiential domain of negative symptoms, everyday life skills, and to a lesser degree, positive symptoms predicted functional outcome after a 4-year follow-up.

The SEM model confirmed that neurocognition, social cognition, positive symptoms, the experiential domain, and available incentives had a significant direct or indirect impact on at least one real-life functioning domain at the 4-year follow-up assessment. Higher baseline neurocognitive functioning predicted better everyday life skills and work skills; better social cognition predicted better work skills and interpersonal relationships; more severe positive symptoms predicted lower work skills; more severe experiential domain symptoms predicted worse interpersonal relationships; and more social incentives predicted better everyday life skills. The LCS model showed that the same baseline variables, except incentives, predicted changes in functioning at the 4-year follow-up. In particular, better baseline neurocognition predicted improvement in everyday life skills, work skills, social cognition, and functional capacity after 4 years. Less severe experiential domain symptoms and better social cognition at baseline predicted improvement in interpersonal relationships at follow-up, while less severe positive symptoms at baseline predicted improvement in work skills. Finally, better baseline everyday life skills predicted improvement in work skills and in functional capacity at follow-up.

The network analysis in the longitudinal study (34) confirmed the results of the cross-sectional one (33). The network structure remained substantially unchanged: neurocognition, social cognition, resilience, and real-life functioning were spatially contiguous and highly interconnected; everyday life skills and functional capacity were the most central and interconnected nodes of the network, while psychopathological domains were more peripheral. The number and the strengths of network connections in non-recovered patients were significantly different compared to those of the recovered ones. In fact, the network of non-recovered patients had more connections, whose strengths were higher than those found in recovered patients. The SLOF domain everyday life skills and disorganization had a higher strength among non-recovered patients, as compared to recovered ones.

### Other Results of the Longitudinal Study

The network longitudinal study also contributed to the investigation of the accuracy of subjects with schizophrenia in self-evaluation of functioning. The study, conducted by Rocca et al. (125), aimed to investigate the concordance of patients' reported impairment in real-life functioning with the caregivers' reported one. Furthermore, it aimed to identify which factors are associated with discrepancies between patients' and caregivers' reports. Results indicated that patients systematically reported a higher functional level than their relatives; however, the patient–caregiver discrepancy was significant only in 17.6% of the cases. The strongest predictors of patient–caregiver discrepancies were caregivers' ratings in each SLOF domain. These findings underline the possibility to use in clinical practice patients' self-evaluation of functioning in order to design tailored rehabilitative programs.

## Electrophysiological and Other Add-On Studies

Two investigations were carried out within the electrophysiological add-on study (126, 127). The first study aimed to investigate neurophysiological correlates of negative symptom domains. This study showed that the brain electrical microstate A (microstate associated with the visual network) was related to the experiential domain and not to the expressive one. Within the experiential domain, avolition, asociality, and anticipatory anhedonia, but not consummatory anhedonia, showed a similar pattern of correlation. These data suggest the existence of distinct electrophysiological correlates of the two negative symptom domains and lend support to the hypothesis that only the anticipatory component of anhedonia shares the same pathophysiological underpinnings of the experiential domain (126). The second study aimed to investigate electrophysiological and neurocognitive correlates of the PANSS disorganization dimension, in order to evaluate the heterogeneity of this dimension and its possible overlap with neurocognitive deficits. The authors reported that the slow alpha activity was negatively correlated with disorganization in subjects with schizophrenia. At item level, only the PANSS item “Difficulty in abstract thinking” showed the same correlation. The MCCB neurocognitive composite score was associated with disorganization dimension as well as PANSS items “Conceptual disorganization” and “Difficulty in abstract thinking”. These findings support a partial overlap between disorganization and neurocognitive impairment. In addition, they suggest that some aspects of disorganization could be related to the impairment of basic neurobiological functions that are only partially evaluated using MCCB (127).

Finally, the network longitudinal study contributed also to the characterization of a subgroup of subjects with schizophrenia defined by the presence of autistic spectrum symptoms. Patients with autistic traits represent a specific population of subject with schizophrenia, characterized by specific patterns of functioning, resilience, and coping abilities (128). Moreover, autistic symptoms may have a relevant impact on different aspects of the disease, in particular neurocognitive and social cognition domains, functional capacity, real-world interpersonal relationships, and participation in everyday life activities (129).

The other add-on studies are still ongoing, and their results have yet to be published.

## Discussion

So far, the network has published more than 20 scientific papers and contributed to the validation of state-of-the-art assessment tools and to the training of many researchers from all the involved centers. Furthermore, in the last decade, the network contributions have led to an improvement in knowledge about main determinants of functioning and, therefore, of clinical recovery in subjects with schizophrenia. Despite the introduction of innovative pharmacological and psychosocial treatments that facilitate symptomatic remission, the impairment in different areas of real-life functioning still represent an unmet need in the care of people suffering from schizophrenia, thus causing a huge burden on patients, their families, and health care systems (31–35, 37, 39–41). The strengths of the two main network studies, with respect to previous studies, include the analysis of a greater number of variables, some of which had never been examined before; the use of state-of-the-art instruments for the assessment of each variable included; and appropriate data analysis methods, in order to explore the complex relationship between possible predictors, mediators, and functional outcome measures. Finally, the implementation of the longitudinal study allowed us to overcome the limitations of the cross-sectional design that prevented inferences about the direction of causality.

The findings from the network studies (32–34, 37) suggest that different factors–some related to the illness, some to personal resources, and others to the social context–contribute to functional outcome, through direct or indirect associations.

The network findings strongly support the implementation of integrated treatments, combining pharmacological, psychosocial, and rehabilitative interventions. In fact, pharmacotherapy is mainly used in order to achieve the remission of positive symptoms, which had a small impact on real-life functioning of subjects with schizophrenia. Functional capacity and everyday life skills were the most central and interconnected nodes of the schizophrenia network, suggesting that they should be the main target of rehabilitative recovery-oriented programs. Moreover, since impairment in neurocognition and social cognition was the most important predictors of real-life functioning, cognitive remediation interventions should be integrated into routine clinical practice. Negative symptoms, in particular those belonging to the experiential domain, i.e., avolition, asociality, and anhedonia, have a direct impact on interpersonal functioning and predict follow-up levels of functioning in the same domain. These negative symptoms do not show any connections with functional capacity or social and non-social cognitive abilities. Their treatment remains an unmet need of schizophrenia care. Further research is needed in order to disentangle the complexity of this negative symptom domain, looking also at behavioral and neurobiological correlates in order to search for effective treatments (130).

The research question concerning the relationships of cognitive impairment and negative symptoms has been investigated with an innovative approach in the two network studies. In the cross-sectional study, SEM analysis demonstrated that only the experiential domain of negative symptoms had a distinct direct effect on functioning, while the expressive domain was not retained in the model when including cognitive impairment. The network analysis findings added further insight to the issue, showing that expressive deficit had no direct connection to real-life functioning nodes and was connected to functional capacity, as were social and non-social cognitive variables, and to disorganization. The experiential domain was directly connected to the interpersonal relationships and work skills domains of real-life functioning, while having no direct or indirect connections with the cognitive nodes, functional capacity, and disorganization. These findings were confirmed by the network analysis carried out on the follow-up data and have implications for the personalized management of patients with negative symptoms. The implementation of psychosocial interventions focused on motivation and pleasure, which targeted the experiential domain of negative symptoms, is very recent and awaits further testing. Effective treatments such as social skills training and cognitive remediation interventions should be made available to subjects with negative symptoms, in particular to those with expressive deficit, such as alogia and blunted affect. Clinical research on the effectiveness of these interventions for negative symptoms should always include at least as a secondary outcome the differential efficacy on the two domains of the negative symptoms.

The network studies document that many other factors have an impact on functional outcome, such as other aspects of psychopathology, personal resources, or context-related factors. This suggests the importance of personalized treatments based on a detailed characterization of each patient, as recently suggested by a group of experts in the field (51).

Moreover, the electrophysiological add-on studies of the network contributed to improve the knowledge of neurophysiological correlates of psychopathology. In fact, although several papers reported resting-state electrophysiological alterations in subjects with schizophrenia (131–133) and their association with psychopathology and cognitive impairment (134, 135), no study investigated the neurophysiological mechanisms underlying distinct negative symptom domains and the disorganization dimension. The network studies showed the existence of distinct electrophysiological correlates of the two negative symptom domains. Furthermore, they suggested the partial overlap between disorganization and neurocognitive impairment and the relationship of some aspects of disorganization with basic electrophysiological alterations which might represent biomarkers of this dimension.

Finally, moving from the evidence of significant levels of autistic traits in a substantial proportion of patients with schizophrenia (136, 137), the network add-on studies on this topic showed that patients with autistic traits represent a population of subject with schizophrenia, characterized by peculiar patterns of social and non-social cognitive impairment and deficits in real-life functioning, thus suggesting that these patients might benefit from specific and targeted interventions.

The network will continue to promote research in this field in order to improve the functional outcome of people suffering from schizophrenia, thus reducing the burden on patients, their families, and health care systems.

## Author Contributions

The project idea was initiated by SG, involving a collaboration with LG and GG. LG wrote the first draft of the manuscript. All authors were responsible for the interpretation of the results, contributed to critically revising the content, and approved the final manuscript for submission to *Frontiers in Psychiatry*.

## Funding

The study was funded by the Italian Ministry of Education (Grant Number: 2010XP2XR4), the Italian Society of Psychopathology (SOPSI), the Italian Society of Biological Psychiatry (SIPB), Roche, Switzerland Lilly, United States AstraZeneca, United Kingdom Lundbeck foundation, Denmark, and Bristol-Myers Squibb, United Kingdom. These entities had no role in the study design, in the collection, analysis, and interpretation of data, in the writing of the report, and in the decision to submit the paper for publication.

## Conflict of Interest

The authors declare that the research was conducted in the absence of any commercial or financial relationships that could be construed as a potential conflict of interest.

## Publisher's Note

All claims expressed in this article are solely those of the authors and do not necessarily represent those of their affiliated organizations, or those of the publisher, the editors and the reviewers. Any product that may be evaluated in this article, or claim that may be made by its manufacturer, is not guaranteed or endorsed by the publisher.

## References

[1] Tandon R Nasrallah HA Keshavan MS. Schizophrenia, “just the facts” 4. Clinical features and conceptualization. Schizophr Res. (2009) 110:1–23. 10.1016/j.schres.2009.03.005 19328655

[2] Guloksuz S Pries LK Delespaul P Kenis G Luykx JJ Lin BD. Examining the independent and joint effects of molecular genetic liability and environmental exposures in schizophrenia: results from the EUGEI study. World Psychiatry. (2019) 18:173–82. 10.1002/wps.20629 31059627 PMC6502485

[3] Bond GR Drake RE Becker DR. An update on individual placement and support. World Psychiatry. (2020) 19:390–1. 10.1002/wps.20784 32931093 PMC7491619

[4] Reichenberg A Velthorst E Davidson M. Cognitive impairment and psychosis in schizophrenia: independent or linked conditions? World Psychiatry. (2019) 18:162–3. 10.1002/wps.20644 31059615 PMC6502409

[5] Reininghaus U Bohnke JR Chavez-Baldini U Gibbons R Ivleva E Clementz BA. Transdiagnostic dimensions of psychosis in the Bipolar-Schizophrenia Network on Intermediate Phenotypes (B-SNIP). World Psychiatry. (2019) 18:67–76. 10.1002/wps.20607 30600629 PMC6313235

[6] Huhn M Nikolakopoulou A Schneider-Thoma J Krause M Samara M Peter N. Comparative efficacy and tolerability of 32 oral antipsychotics for the acute treatment of adults with multi-episode schizophrenia: a systematic review and network meta-analysis. Lancet. (2019) 394:939–51. 10.1016/S0140-6736(19)31135-3 31303314 PMC6891890

[7] Kishimoto T Hagi K Nitta M Kane JM Correll CU. Long-term effectiveness of oral second-generation antipsychotics in patients with schizophrenia and related disorders: a systematic review and meta-analysis of direct head-to-head comparisons. World Psychiatry. (2019) 18:208–24. 10.1002/wps.20632 31059621 PMC6502423

[8] McKenna P Leucht S Jauhar S Laws K Bighelli I. The controversy about cognitive behavioural therapy for schizophrenia. World Psychiatry. (2019) 18:235–6. 10.1002/wps.20636 31059624 PMC6502403

[9] Reed GM First MB Kogan CS Hyman SE Gureje O Gaebel W. Innovations and changes in the ICD-11 classification of mental, behavioural and neurodevelopmental disorders. World Psychiatry. (2019) 18:3–19. 10.1002/wps.20611 30600616 PMC6313247

[10] Gaebel W Falkai P Hasan A. The revised German evidence- and consensus-based schizophrenia guideline. World Psychiatry. (2020) 19:117–9. 10.1002/wps.20706 31922675 PMC6953579

[11] Guloksuz S Pries LK Ten Have M de Graaf R van Dorsselaer S Klingenberg B. Association of preceding psychosis risk states and non-psychotic mental disorders with incidence of clinical psychosis in the general population: a prospective study in the NEMESIS-2 cohort. World Psychiatry. (2020) 19:199–205. 10.1002/wps.20755 32394548 PMC7215054

[12] Kotov R Jonas KG Carpenter WT Dretsch MN Eaton NR Forbes MK. Validity and utility of Hierarchical Taxonomy of Psychopathology (HiTOP): I. Psychosis superspectrum. World Psychiatry. (2020) 19:151–72. 10.1002/wps.20730 32394571 PMC7214958

[13] McCutcheon RA Krystal JH Howes OD. Dopamine and glutamate in schizophrenia: biology, symptoms and treatment. World Psychiatry. (2020) 19:15–33. 10.1002/wps.20693 31922684 PMC6953551

[14] McCutcheon RA Reis Marques T Howes OD. Schizophrenia-an overview. JAMA Psychiatry. (2020) 77:201–10. 10.1001/jamapsychiatry.2019.3360 31664453

[15] Moritz S Silverstein SM Dietrichkeit M Gallinat J. Neurocognitive deficits in schizophrenia are likely to be less severe and less related to the disorder than previously thought. World Psychiatry. (2020) 19:254–5. 10.1002/wps.20759 32394552 PMC7215075

[16] Singh SP Javed A. Psychosis WPAEIAPfEIi. Early intervention in psychosis in low- and middle-income countries: a WPA initiative. World Psychiatry. (2020) 19:122. 10.1002/wps.20708 31922694 PMC6953594

[17] Smeland OB Frei O Dale AM Andreassen OA. The polygenic architecture of schizophrenia - rethinking pathogenesis and nosology. Nat Rev Neurol. (2020) 16:366–79. 10.1038/s41582-020-0364-0 32528109

[18] Taipale H Tanskanen A Mehtala J Vattulainen P Correll CU Tiihonen J. 20-year follow-up study of physical morbidity and mortality in relationship to antipsychotic treatment in a nationwide cohort of 62,250 patients with schizophrenia (FIN20). World Psychiatry. (2020) 19:61–8. 10.1002/wps.20699 31922669 PMC6953552

[19] Zandersen M Parnas J. Borderline personality disorder or a disorder within the schizophrenia spectrum? A psychopathological study. World Psychiatry. (2019) 18:109–10. 10.1002/wps.20598 30600641 PMC6313234

[20] Andreasen NC Carpenter WT Jr Kane JM Lasser RA Marder SR Weinberger DR. Remission in schizophrenia: proposed criteria and rationale for consensus. Am J Psychiatry. (2005) 162:441–9. 10.1176/appi.ajp.162.3.441 15741458

[21] Bebbington PE Craig T Garety P Fowler D Dunn G Colbert S. Remission and relapse in psychosis: operational definitions based on case-note data. Psychol Med. (2006) 36:1551–62. 10.1017/S0033291706008579 16911809

[22] Haro JM Novick D Suarez D Alonso J Lepine JP Ratcliffe M. Remission and relapse in the outpatient care of schizophrenia: three-year results from the Schizophrenia Outpatient Health Outcomes study. J Clin Psychopharmacol. (2006) 26:571–8. 10.1097/01.jcp.0000246215.49271.b8 17110813

[23] Vita A Barlati S. Recovery from schizophrenia: is it possible? Curr Opin Psychiatry. (2018) 31:246–55. 10.1097/YCO.0000000000000407 29474266

[24] Jaaskelainen E Juola P Hirvonen N McGrath JJ Saha S Isohanni M. A systematic review and meta-analysis of recovery in schizophrenia. Schizophr Bull. (2013) 39:1296–306. 10.1093/schbul/sbs130 23172003 PMC3796077

[25] Liberman RP Kopelowicz A. Recovery from schizophrenia: a concept in search of research. Psychiatr Serv. (2005) 56:735–42. 10.1176/appi.ps.56.6.735 15939952

[26] Davidson M. Cognitive impairment as a diagnostic criterion and treatment target in schizophrenia. World Psychiatry. (2019) 18:171–2. 10.1002/wps.20651 31059612 PMC6502436

[27] Harvey PD Bellack AS. Toward a terminology for functional recovery in schizophrenia: is functional remission a viable concept? Schizophr Bull. (2009) 35:300–6. 10.1093/schbul/sbn171 19126634 PMC2659311

[28] Harvey PD Strassnig MT. Cognition and disability in schizophrenia: cognition-related skills deficits and decision-making challenges add to morbidity. World Psychiatry. (2019) 18:165–7. 10.1002/wps.20647 31059625 PMC6502430

[29] Shrivastava A Johnston M Shah N Bureau Y. Redefining outcome measures in schizophrenia: integrating social and clinical parameters. Curr Opin Psychiatry. (2010) 23:120–6. 10.1097/YCO.0b013e328336662e 20057314

[30] Falkai P Schmitt A. The need to develop personalized interventions to improve cognition in schizophrenia. World Psychiatry. (2019) 18:170. 10.1002/wps.20650 31059619 PMC6502408

[31] Fleischhacker WW Arango C Arteel P Barnes TR Carpenter W Duckworth K. Schizophrenia–time to commit to policy change. Schizophr Bull. (2014) 40 Suppl 3:S165–94. 10.1093/schbul/sbu006 24778411 PMC4002061

[32] Galderisi S Rossi A Rocca P Bertolino A Mucci A Bucci P. The influence of illness-related variables, personal resources and context-related factors on real-life functioning of people with schizophrenia. World Psychiatry. (2014) 13:275–87. 10.1002/wps.20167 25273301 PMC4219069

[33] Galderisi S Rucci P Kirkpatrick B Mucci A Gibertoni D Rocca P. Interplay among psychopathologic variables, personal resources, context-related factors, and real-life functioning in individuals with schizophrenia: a network analysis. JAMA Psychiatry. (2018) 75:396–404. 10.1001/jamapsychiatry.2017.4607 29450447 PMC5875306

[34] Galderisi S Rucci P Mucci A Rossi A Rocca P Bertolino A. The interplay among psychopathology, personal resources, context-related factors and real-life functioning in schizophrenia: stability in relationships after 4 years and differences in network structure between recovered and non-recovered patients. World Psychiatry. (2020) 19:81–91. 10.1002/wps.20700 31922687 PMC6953544

[35] Green MF Hellemann G Horan WP Lee J Wynn JK. From perception to functional outcome in schizophrenia: modeling the role of ability and motivation. Arch Gen Psychiatry. (2012) 69:1216–24. 10.1001/archgenpsychiatry.2012.652 23026889 PMC3976993

[36] Keefe RSE. Why are there no approved treatments for cognitive impairment in schizophrenia? World Psychiatry. (2019) 18:167–8. 10.1002/wps.20648 31059617 PMC6502426

[37] Mucci A Galderisi S Gibertoni D Rossi A Rocca P Bertolino A. Factors associated with real-life functioning in persons with schizophrenia in a 4-year follow-up study of the Italian Network for Research on Psychoses. JAMA Psychiatry. (2021) 78:550–9. 33566071 10.1001/jamapsychiatry.2020.4614 PMC7876615

[38] Sahakian BJ Savulich G. Innovative methods for improving cognition, motivation and wellbeing in schizophrenia. World Psychiatry. (2019) 18:168–70. 10.1002/wps.20649 31059610 PMC6502422

[39] Strassnig MT Raykov T O'Gorman C Bowie CR Sabbag S Durand D. Determinants of different aspects of everyday outcome in schizophrenia: the roles of negative symptoms, cognition, and functional capacity. Schizophr Res. (2015) 165:76–82. 10.1016/j.schres.2015.03.033 25868935 PMC4437911

[40] Ventura J Subotnik KL Gitlin MJ Gretchen-Doorly D Ered A Villa KF. Negative symptoms and functioning during the first year after a recent onset of schizophrenia and 8 years later. Schizophr Res. (2015) 161:407–13. 10.1016/j.schres.2014.10.043 25499044 PMC4308531

[41] Leifker FR Bowie CR Harvey PD. Determinants of everyday outcomes in schizophrenia: the influences of cognitive impairment, functional capacity, and symptoms. Schizophr Res. (2009) 115:82–7. 10.1016/j.schres.2009.09.004 19775869

[42] Bowie CR Reichenberg A Patterson TL Heaton RK Harvey PD. Determinants of real-world functional performance in schizophrenia subjects: correlations with cognition, functional capacity, and symptoms. Am J Psychiatry. (2006) 163:418–25. 10.1176/appi.ajp.163.3.418 16513862

[43] Drake RE Xie H McHugo GJ. A 16-year follow-up of patients with serious mental illness and co-occurring substance use disorder. World Psychiatry. (2020) 19:397–8. 10.1002/wps.20793 32931112 PMC7491638

[44] Green MF Horan WP Lee J. Nonsocial and social cognition in schizophrenia: current evidence and future directions. World Psychiatry. (2019) 18:146–61. 10.1002/wps.20624 31059632 PMC6502429

[45] Harvey PD. Functional recovery in schizophrenia: raising the bar for outcomes in people with schizophrenia. Schizophr Bull. (2009) 35:299. 10.1093/schbul/sbn186 19244591 PMC2659319

[46] Ho BC Nopoulos P Flaum M Arndt S Andreasen NC. Two-year outcome in first-episode schizophrenia: predictive value of symptoms for quality of life. Am J Psychiatry. (1998) 155:1196–201. 10.1176/ajp.155.9.1196 9734542

[47] Hultman CM Wieselgren IM Ohman A. Relationships between social support, social coping and life events in the relapse of schizophrenic patients. Scand J Psychol. (1997) 38:3–13. 10.1111/1467-9450.00002 9104101

[48] Livingston JD Boyd JE. Correlates and consequences of internalized stigma for people living with mental illness: a systematic review and meta-analysis. Soc Sci Med. (2010) 71:2150–61. 10.1016/j.socscimed.2010.09.030 21051128

[49] Torgalsboen AK. Sustaining full recovery in schizophrenia after 15 years: does resilience matter? Clin Schizophr Relat Psychoses. (2012) 5:193–200. 10.3371/CSRP.5.4.3 22182456

[50] Ventura J Hellemann GS Thames AD Koellner V Nuechterlein KH. Symptoms as mediators of the relationship between neurocognition and functional outcome in schizophrenia: a meta-analysis. Schizophr Res. (2009) 113:189–99. 10.1016/j.schres.2009.03.035 19628375 PMC2825750

[51] Maj M van Os J De Hert M Gaebel W Galderisi S Green MF. The clinical characterization of the patient with primary psychosis aimed at personalization of management. World Psychiatry. (2021) 20:4–33. 10.1002/wps.20809 33432763 PMC7801854

[52] Vancampfort D Firth J Correll CU Solmi M Siskind D De Hert M. The impact of pharmacological and non-pharmacological interventions to improve physical health outcomes in people with schizophrenia: a meta-review of meta-analyses of randomized controlled trials. World Psychiatry. (2019) 18:53–66. 10.1002/wps.20614 30600626 PMC6313230

[53] Ahmed AO Murphy CF Latoussakis V McGovern KE English J Bloch A. An examination of neurocognition and symptoms as predictors of post-hospital community tenure in treatment resistant schizophrenia. Psychiatry Res. (2016) 236:47–52. 10.1016/j.psychres.2016.01.001 26778628

[54] Chang WC Hui CL Chan SK Lee EH Chen EY. Impact of avolition and cognitive impairment on functional outcome in first-episode schizophrenia-spectrum disorder: a prospective one-year follow-up study. Schizophr Res. (2016) 170:318–21. 10.1016/j.schres.2016.01.004 26778673

[55] Fu S Czajkowski N Rund BR Torgalsboen AK. The relationship between level of cognitive impairments and functional outcome trajectories in first-episode schizophrenia. Schizophr Res. (2017) 190:144–9. 10.1016/j.schres.2017.03.002 28302394

[56] Galderisi S Bucci P Mucci A Kirkpatrick B Pini S Rossi A. Categorical and dimensional approaches to negative symptoms of schizophrenia: focus on long-term stability and functional outcome. Schizophr Res. (2013) 147:157–62. 10.1016/j.schres.2013.03.020 23608244

[57] Galderisi S Mucci A Bitter I Libiger J Bucci P Fleischhacker WW. Persistent negative symptoms in first episode patients with schizophrenia: results from the European First Episode Schizophrenia Trial. Eur Neuropsychopharmacol. (2013) 23:196–204. 10.1016/j.euroneuro.2012.04.019 22647933

[58] Green MF Kern RS Heaton RK. Longitudinal studies of cognition and functional outcome in schizophrenia: implications for MATRICS. Schizophr Res. (2004) 72:41–51. 10.1016/j.schres.2004.09.009 15531406

[59] Horan WP Green MF DeGroot M Fiske A Hellemann G Kee K. Social cognition in schizophrenia, Part 2: 12-month stability and prediction of functional outcome in first-episode patients. Schizophr Bull. (2012) 38:865–72. 10.1093/schbul/sbr001 21382881 PMC3406537

[60] McCleery A Lee J Fiske AP Ghermezi L Hayata JN Hellemann GS. Longitudinal stability of social cognition in schizophrenia: a 5-year follow-up of social perception and emotion processing. Schizophr Res. (2016) 176:467–72. 10.1016/j.schres.2016.07.008 27443808 PMC5026923

[61] Reichenberg A Feo C Prestia D Bowie CR Patterson TL Harvey PD. The course and correlates of everyday functioning in schizophrenia. Schizophr Res Cogn. (2014) 1:e47–52. 10.1016/j.scog.2014.03.001 25045625 PMC4097820

[62] Robinson DG Woerner MG McMeniman M Mendelowitz A Bilder RM. Symptomatic and functional recovery from a first episode of schizophrenia or schizoaffective disorder. Am J Psychiatry. (2004) 161:473–9. 10.1176/appi.ajp.161.3.473 14992973

[63] Stirling J White C Lewis S Hopkins R Tantam D Huddy A. Neurocognitive function and outcome in first-episode schizophrenia: a 10-year follow-up of an epidemiological cohort. Schizophr Res. (2003) 65:75–86. 10.1016/S0920-9964(03)00014-8 14630300

[64] Daniel DG. Issues in selection of instruments to measure negative symptoms. Schizophr Res. (2013) 150:343–5. 10.1016/j.schres.2013.07.005 23899996

[65] Kern RS Nuechterlein KH Green MF Baade LE Fenton WS Gold JM. The MATRICS consensus cognitive battery, part 2: co-norming and standardization. Am J Psychiatry. (2008) 165:214–20. 10.1176/appi.ajp.2007.07010043 18172018

[66] Kirkpatrick B Strauss GP Nguyen L Fischer BA Daniel DG Cienfuegos A. The brief negative symptom scale: psychometric properties. Schizophr Bull. (2011) 37:300–5. 10.1093/schbul/sbq059 20558531 PMC3044634

[67] Nuechterlein KH Green MF Kern RS Baade LE Barch DM Cohen JD. The MATRICS consensus cognitive battery, part 1: test selection, reliability, and validity. Am J Psychiatry. (2008) 165:203–13. 10.1176/appi.ajp.2007.07010042 18172019

[68] Grant PM Best MW Beck AT. The meaning of group differences in cognitive test performance. World Psychiatry. (2019) 18:163–4. 10.1002/wps.20645 31059607 PMC6502459

[69] Melle I. Cognition in schizophrenia: a marker of underlying neurodevelopmental problems? World Psychiatry. (2019) 18:164–5. 10.1002/wps.20646 31059634 PMC6502404

[70] Mucci A Vignapiano A Bitter I Austin SF Delouche C Dollfus S. A large European, multicenter, multinational validation study of the Brief Negative Symptom Scale. Eur Neuropsychopharm. (2019) 29:947–59. 10.1016/j.euroneuro.2019.05.006 31255394

[71] Couture SM Penn DL Roberts DL. The functional significance of social cognition in schizophrenia: a review. Schizophr Bull. (2006) 32(Suppl 1):S44–63. 10.1093/schbul/sbl029 16916889 PMC2632537

[72] Green MF Leitman DI. Social cognition in schizophrenia. Schizophr Bull. (2008) 34:670–2. 10.1093/schbul/sbn045 18495642 PMC2632454

[73] Green MF. What are the functional consequences of neurocognitive deficits in schizophrenia? Am J Psychiatry. (1996) 153:321–30. 10.1176/ajp.153.3.321 8610818

[74] Fett AK Viechtbauer W Dominguez MD Penn DL van Os J Krabbendam L. The relationship between neurocognition and social cognition with functional outcomes in schizophrenia: a meta-analysis. Neurosci Biobehav Rev. (2011) 35:573–88. 10.1016/j.neubiorev.2010.07.001 20620163

[75] Kee KS Green MF Mintz J Brekke JS. Is emotion processing a predictor of functional outcome in schizophrenia? Schizophr Bull. (2003) 29:487–97. 10.1093/oxfordjournals.schbul.a007021 14609242

[76] Galderisi S Mucci A Buchanan RW Arango C. Negative symptoms of schizophrenia: new developments and unanswered research questions. Lancet Psychiatry. (2018) 5:664–77. 10.1016/S2215-0366(18)30050-6 29602739

[77] Couture SM Granholm EL Fish SC. A path model investigation of neurocognition, theory of mind, social competence, negative symptoms and real-world functioning in schizophrenia. Schizophr Res. (2011) 125:152–60. 10.1016/j.schres.2010.09.020 20965699 PMC3031755

[78] Bowie CR Leung WW Reichenberg A McClure MM Patterson TL Heaton RK. Predicting schizophrenia patients' real-world behavior with specific neuropsychological and functional capacity measures. Biol Psychiatry. (2008) 63:505–11. 10.1016/j.biopsych.2007.05.022 17662256 PMC2335305

[79] Blanchard JJ Cohen AS. The structure of negative symptoms within schizophrenia: implications for assessment. Schizophr Bull. (2006) 32:238–45. 10.1093/schbul/sbj013 16254064 PMC2632211

[80] Kirkpatrick B Fischer B. Subdomains within the negative symptoms of schizophrenia: commentary. Schizophr Bull. (2006) 32:246–9. 10.1093/schbul/sbj054 16492798 PMC2632226

[81] Strauss GP Esfahlani FZ Galderisi S Mucci A Rossi A Bucci P. Network analysis reveals the latent structure of negative symptoms in schizophrenia. Schizophr Bull. (2019) 45:1033–41. 10.1093/schbul/sby133 30256991 PMC6737465

[82] Rieckmann N Reichenberg A Bowie CR Parrella M White L Friedman JI. Depressed mood and its functional correlates in institutionalized schizophrenia patients. Schizophr Res. (2005) 77:179–87. 10.1016/j.schres.2005.04.007 15894461

[83] Best MW Gupta M Bowie CR Harvey PD. A longitudinal examination of the moderating effects of symptoms on the relationship between functional competence and real world functional performance in schizophrenia. Schizophr Res Cogn. (2014) 1:90–5. 10.1016/j.scog.2014.03.002 25267939 PMC4175398

[84] Pogue-Geile MF Harrow M. Negative and positive symptoms in schizophrenia and depression: a followup. Schizophr Bull. (1984) 10:371–87. 10.1093/schbul/10.3.371 6474100

[85] Norman RM Malla AK Cortese L Cheng S Diaz K McIntosh E. Symptoms and cognition as predictors of community functioning: a prospective analysis. Am J Psychiatry. (1999) 156:400–5. 10080555 10.1176/ajp.156.3.400

[86] Tait L Birchwood M Trower P. Adapting to the challenge of psychosis: personal resilience and the use of sealing-over (avoidant) coping strategies. Br J Psychiatry. (2004) 185:410–5. 10.1192/bjp.185.5.410 15516550

[87] Tait L Birchwood M Trower P. A new scale (SES) to measure engagement with community mental health services. J Ment Health. (2002) 11:191–8. 10.1080/09638230020023570-2 21208145

[88] Gerlinger G Hauser M De Hert M Lacluyse K Wampers M Correll CU. Personal stigma in schizophrenia spectrum disorders: a systematic review of prevalence rates, correlates, impact and interventions. World Psychiatry. (2013) 12:155–64. 10.1002/wps.20040 23737425 PMC3683268

[89] Kay SR Fiszbein A Opler LA. The positive and negative syndrome scale (PANSS) for schizophrenia. Schizophr Bull. (1987) 13:261–76. 10.1093/schbul/13.2.261 3616518

[90] Galderisi S Mucci A Dollfus S Nordentoft M Falkai P Kaiser S. EPA guidance on assessment of negative symptoms in schizophrenia. Eur Psychiatry. (2021) 64:e23. 10.1192/j.eurpsy.2021.11 33597064 PMC8080207

[91] Garcia-Portilla MP Garcia-Alvarez L Saiz PA Al-Halabi S Bobes-Bascaran MT Bascaran MT. Psychometric evaluation of the negative syndrome of schizophrenia. Eur Arch Psychiatry Clin Neurosci. (2015) 265:559–66. 10.1007/s00406-015-0595-z 25802109

[92] Mucci A Galderisi S Merlotti E Rossi A Rocca P Bucci P. The Brief Negative Symptom Scale (BNSS): Independent validation in a large sample of Italian patients with schizophrenia. Eur Psychiatry. (2015) 30:641–7. 10.1016/j.eurpsy.2015.01.014 25758156

[93] Addington D Addington J Maticka-Tyndale E. Assessing depression in schizophrenia: the Calgary Depression Scale. Br J Psychiatry Suppl. (1993) 22:39–44. 10.1192/S0007125000292581 8110442

[94] Gerlach J Korsgaard S Clemmesen P Lauersen AM Magelund G Noring U. The St. Hans Rating Scale for extrapyramidal syndromes: reliability and validity. Acta Psychiatr Scand. (1993) 87:244–52. 10.1111/j.1600-0447.1993.tb03366.x 8098178

[95] Kerr SL Neale JM. Emotion perception in schizophrenia: specific deficit or further evidence of generalized poor performance? J Abnorm Psychol. (1993) 102:312–8. 10.1037/0021-843X.102.2.312 8315144

[96] McDonald S Bornhofen C Shum D Long E Saunders C Neulinger K. Reliability and validity of The Awareness of Social Inference Test (TASIT): a clinical test of social perception. Disabil Rehabil. (2006) 28:1529–42. 10.1080/09638280600646185 17178616

[97] Friborg O Hjemdal O Rosenvinge JH Martinussen M. A new rating scale for adult resilience: what are the central protective resources behind healthy adjustment? Int J Methods Psychiatr Res. (2003) 12:65–76. 10.1002/mpr.143 12830300 PMC6878238

[98] Ritsher JB Otilingam PG Grajales M. Internalized stigma of mental illness: psychometric properties of a new measure. Psychiatry Res. (2003) 121:31–49. 10.1016/j.psychres.2003.08.008 14572622

[99] Mausbach BT Harvey PD Goldman SR Jeste DV Patterson TL. Development of a brief scale of everyday functioning in persons with serious mental illness. Schizophr Bull. (2007) 33:1364–72. 10.1093/schbul/sbm014 17341468 PMC2779885

[100] Mucci A Rucci P Rocca P Bucci P Gibertoni D Merlotti E. The specific level of functioning scale: construct validity, internal consistency and factor structure in a large Italian sample of people with schizophrenia living in the community. Schizophr Res. (2014) 159:144–50. 10.1016/j.schres.2014.07.044 25182540

[101] Schneider LC Struening EL. SLOF a behavioral rating scale for assessing the mentally ill. Soc Work Res Abstr. (1983) 19:9–21. 10.1093/swra/19.3.9 10264257

[102] Harvey PD Raykov T Twamley EW Vella L Heaton RK Patterson TL. Validating the measurement of real-world functional outcomes: phase I results of the VALERO study. Am J Psychiatry. (2011) 168:1195–201. 10.1176/appi.ajp.2011.10121723 21572166 PMC3670945

[103] Leifker FR Patterson TL Heaton RK Harvey PD. Validating measures of real-world outcome: the results of the VALERO expert survey and RAND panel. Schizophr Bull. (2011) 37:334–43. 10.1093/schbul/sbp044 19525354 PMC3044614

[104] Merlotti E Mucci A Bucci P Nardi A Galderisi S. Italian version of the “Brief Negative Symptom Scale”. J Psychopathol. (2015) 20:199–215.

[105] Montemagni C Rocca P Mucci A Galderisi S Maj M. Italian version of the “Specific Level of Functioning”. J Psychopathol. (2015) 21:287–96.

[106] Mancuso F Mucci A Galderisi S. MATRICS consensus cognitive battery: storia e sviluppo della batteria cognitiva. Nòos - Aggiornamenti Psichiatria. (2013) 19:83–98. 10.1722/3349.33203

[107] Mucci A Galderisi S Green MF Nuechterlein K Rucci P Gibertoni D. Familial aggregation of MATRICS Consensus Cognitive Battery scores in a large sample of outpatients with schizophrenia and their unaffected relatives. Psychol Med. (2018) 48:1359–66. 10.1017/S0033291717002902 29017620

[108] Rocca P Galderisi S Rossi A Bertolino A Rucci P Gibertoni D. Social cognition in people with schizophrenia: a cluster-analytic approach. Psychol Med. (2016) 46:2717–29. 10.1017/S0033291716001100 27649341

[109] Beran TN Violato C. Structural equation modeling in medical research: a primer. BMC Res Notes. (2010) 3:267. 10.1186/1756-0500-3-267 20969789 PMC2987867

[110] Forbes MK Wright AGC Markon KE Krueger RF. The network approach to psychopathology: promise versus reality. World Psychiatry. (2019) 18:272–3. 10.1002/wps.20659 31496101 PMC6732676

[111] Hevey D. Network analysis: a brief overview and tutorial. Health Psychol Behav Med. (2018) 6:301–28. 10.1080/21642850.2018.1521283 34040834 PMC8114409

[112] Galderisi S Rossi A Rocca P Bertolino A Mucci A Bucci P. Pathways to functional outcome in subjects with schizophrenia living in the community and their unaffected first-degree relatives. Schizophr Res. (2016) 175:154–60. 10.1016/j.schres.2016.04.043 27209527

[113] Rossi A Amore M Galderisi S Rocca P Bertolino A Aguglia E. The complex relationship between self-reported 'personal recovery' and clinical recovery in schizophrenia. Schizophr Res. (2018) 192:108–12. 10.1016/j.schres.2017.04.040 28495492

[114] Rossi A Galderisi S Rocca P Bertolino A Mucci A Rucci P. The relationships of personal resources with symptom severity and psychosocial functioning in persons with schizophrenia: results from the Italian Network for Research on Psychoses study. Eur Arch Psychiatry Clin Neurosci. (2017) 267:285–94. 10.1007/s00406-016-0710-9 27381016

[115] Rossi A Galderisi S Rocca P Bertolino A Rucci P Gibertoni D. Personal resources and depression in schizophrenia: the role of self-esteem, resilience and internalized stigma. Psychiatry Res. (2017) 256:359–64. 10.1016/j.psychres.2017.06.079 28686933

[116] Rocca P Galderisi S Rossi A Bertolino A Rucci P Gibertoni D. Disorganization and real-world functioning in schizophrenia: results from the multicenter study of the Italian Network for Research on Psychoses. Schizophr Res. (2018) 201:105–12. 10.1016/j.schres.2018.06.003 29898819

[117] Bucci P Galderisi S Mucci A Rossi A Rocca P Bertolino A. Premorbid academic and social functioning in patients with schizophrenia and its associations with negative symptoms and cognition. Acta Psychiatr Scand. (2018) 138:253–66. 10.1111/acps.12938 29984409

[118] Bowie CR. Cognitive remediation for severe mental illness: state of the field and future directions. World Psychiatry. (2019) 18:274–5. 10.1002/wps.20660 31496075 PMC6732698

[119] Amore M Murri MB Calcagno P Rocca P Rossi A Aguglia E. The association between insight and depressive symptoms in schizophrenia: Undirected and Bayesian network analyses. Eur Psychiatry. (2020) 63:1–21. 10.1192/j.eurpsy.2020.45 32372731 PMC7358633

[120] Monteleone P Cascino G Monteleone AM Rocca P Rossi A Bertolino A. Prevalence of antipsychotic-induced extrapyramidal symptoms and their association with neurocognition and social cognition in outpatients with schizophrenia in the “real-life”. Prog Neuropsychopharmacol Biol Psychiatry. (2021) 109:110250. 10.1016/j.pnpbp.2021.110250 33484755

[121] Gennarelli M Monteleone P Minelli A Monteleone AM Rossi A Rocca P. Genome-wide association study detected novel susceptibility genes for social cognition impairment in people with schizophrenia. World J Biol Psychiatry. (2021) 1–9. 10.1080/15622975.2021.1907722 [Epub ahead of print]. 34132174

[122] Piluso G Monteleone P Galderisi S Giugliano T Bertolino A Rocca P. Assessment of *de novo* copy-number variations in Italian patients with schizophrenia: detection of putative mutations involving regulatory enhancer elements. World J Biol Psychiatry. (2019) 20:126–36. 10.1080/15622975.2017.1395072 29069978

[123] Ventura J Reise SP Keefe RS Baade LE Gold JM Green MF. The Cognitive Assessment Interview (CAI): development and validation of an empirically derived, brief interview-based measure of cognition. Schizophr Res. (2010) 121:24–31. 10.1016/j.schres.2010.04.016 20542412 PMC3184638

[124] Palumbo D Bucci P Mucci A Pietrafesa D Giordano GM Vignapiano A. Inter-rater reliability and psychometric characteristics of the Italian version of the Cognitive Assessment Interview (CAI). J Psychopathol. (2019) 25:85–114.

[125] Rocca P Brasso C Montemagni C Bellino S Rossi A Bertolino A. Accuracy of self-assessment of real-life functioning in schizophrenia. NPJ Schizophr. (2021) 7:11. 10.1038/s41537-021-00140-9 33589645 PMC7884703

[126] Giordano GM Koenig T Mucci A Vignapiano A Amodio A Di Lorenzo G. Neurophysiological correlates of Avolition-apathy in schizophrenia: A resting-EEG microstates study. Neuroimage Clin. (2018) 20:627–36. 10.1016/j.nicl.2018.08.031 30202724 PMC6128100

[127] Vignapiano A Mucci A Giordano GM Di Lorenzo G Ferrentino F Altamura M. Neurophysiological correlates of negative symptom domains: an auditory oddball study in schizophrenia. Eur Psychiat. (2019) 56:S287–S8. 10.1016/S0924-977X(16)31527-9

[128] Dell'Osso L Carpita B Cremone IM Gesi C D'Ermo A De Iorio G. Autism spectrum in patients with schizophrenia: correlations with real-life functioning, resilience, and coping styles. CNS Spectr. (2021) 1–11. 10.1017/S1092852921000353 [Epub ahead of print]. 33843551

[129] Vita A Barlati S Deste G Rocca P Rossi A Bertolino A. The influence of autistic symptoms on social and non-social cognition and on real-life functioning in people with schizophrenia: Evidence from the Italian Network for Research on Psychoses multicenter study. Eur Psychiatry. (2020) 63:e98. 10.1192/j.eurpsy.2020.99 33168115 PMC7737172

[130] Cohen AS Schwartz E Le TP Fedechko T Kirkpatrick B Strauss GP. Using biobehavioral technologies to effectively advance research on negative symptoms. World Psychiatry. (2019) 18:103–4. 10.1002/wps.20593 30600611 PMC6313243

[131] Newson JJ Thiagarajan TC. EEG Frequency Bands in Psychiatric Disorders: A Review of Resting State Studies. Front Hum Neurosci. (2018) 12:521. 10.3389/fnhum.2018.00521 30687041 PMC6333694

[132] da Cruz JR Favrod O Roinishvili M Chkonia E Brand A Mohr C. EEG microstates are a candidate endophenotype for schizophrenia. Nat Commun. (2020) 11:3089. 10.1038/s41467-020-16914-1 32555168 PMC7303216

[133] Galderisi S Mucci A Volpe U Boutros N. Evidence-based medicine and electrophysiology in schizophrenia. Clin EEG Neurosci. (2009) 40:62–77. 10.1177/155005940904000206 19534300

[134] Grohn C Norgren E Eriksson L. A systematic review of the neural correlates of multisensory integration in schizophrenia. Schizophr Res Cogn. (2022) 27:100219. 10.1016/j.scog.2021.100219 34660211 PMC8502765

[135] Li X Honda S Nakajima S Wada M Yoshida K Daskalakis ZJ. TMS-EEG research to elucidate the pathophysiological neural bases in patients with schizophrenia: a systematic review. J Pers Med. (2021) 11:388. 10.3390/jpm11050388 34068580 PMC8150818

[136] Zheng Z Zheng P Zou X. Association between schizophrenia and autism spectrum disorder: A systematic review and meta-analysis. Autism Res. (2018) 11:1110–9. 10.1002/aur.1977 30284394

[137] Kastner A Begemann M Michel TM Everts S Stepniak B Bach C. Autism beyond diagnostic categories: characterization of autistic phenotypes in schizophrenia. BMC Psychiatry. (2015) 15:115. 10.1186/s12888-015-0494-x 25968177 PMC4436160

